# Development of a Recombinant Thermostable Newcastle Disease Virus (NDV) Vaccine Express Infectious Bronchitis Virus (IBV) Multiple Epitopes for Protecting against IBV and NDV Challenges

**DOI:** 10.3390/vaccines8040564

**Published:** 2020-10-01

**Authors:** Lei Tan, Guoyuan Wen, Yanmei Yuan, Meizhen Huang, Yingjie Sun, Ying Liao, Cuiping Song, Weiwei Liu, Yonghong Shi, Huabin Shao, Xusheng Qiu, Chan Ding

**Affiliations:** 1 Department of Avian Diseases, Shanghai Veterinary Research Institute, Chinese Academy of Agricultural Sciences, Shanghai 200241, China; tanlei@shvri.ac.cn (L.T.); yuanyanmei@shvri.ac.cn (Y.Y.); meizhen_huang@163.com (M.H.); sunyingjie@shvri.ac.cn (Y.S.); liaoying@shvri.ac.cn (Y.L.); scp@shvri.ac.cn (C.S.); liuweiwei@shvri.ac.cn (W.L.); shiyonghong@shvri.ac.cn (Y.S.); xsqiu1981@shvri.ac.cn (X.Q.); 2 Institute of Animal Husbandry and Veterinary Sciences, Hubei Academy of Agricultural Sciences, Wuhan 430070, China; wgy_524@163.com (G.W.); shhb1961@163.com (H.S.); 3 Jiangsu Co-Innovation Center for Prevention and Control of Important Animal Infectious Diseases and Zoonoses, Yangzhou 225009, China

**Keywords:** Newcastle disease virus, thermostable, infectious bronchitis virus, multiple epitopes, vaccine

## Abstract

Newcastle disease (ND) and infectious bronchitis (IB) are two highly contagious diseases that severely threaten the poultry industry. The goal of this study is to prevent these two diseases and reduce the vaccine costs during storage and transportation. In this study, we design a thermostable recombinant Newcastle disease virus (NDV) candidate live vaccine strain designated as rLS-T-HN-T/B, which expresses the multiple epitope cassette of the identified infectious bronchitis virus (IBV) (S-T/B). The rLS-T-HN-T/B strain was found to possess similar growth kinetics, passage stability, morphological characteristics, and virulence to the parental LaSota strain. After incubation at 56 °C at the indicated time points, the rLS-T-HN-T/B strain was determined by the hemagglutination (HA), and 50% embryo infectious dose (EID_50_) assays demonstrated that it accords with the criteria for thermostability. The thermostable rLS-T-HN-T/B and parental LaSota vaccines were stored at 25 °C for 16 days prior to immunizing the one-day-old specific pathogen-free (SPF) chicks. Three weeks postimmunization, the virus challenge results suggested that the chicks vaccinated with the rLS-T-HN-T/B vaccine were protected by 100% and 90% against a lethal dose of NDV and IBV, respectively. Furthermore, the trachea ciliary activity assay indicated that the mean ciliostasis score of the chicks vaccinated with thermostable rLS-T-HN-T/B vaccine was significantly superior to that of the LaSota and PBS groups (*p* < 0.05). The rLS-T-HN-T/B vaccine stored at 25 °C for 16 days remained capable of eliciting the immune responses and protecting against IBV and NDV challenges. However, the same storage conditions had a great impact on the parental LaSota strain vaccinated chicks, and the NDV challenge protection ratio was only 20%. We conclude that the thermostable rLS-T-HN-T/B strain is a hopeful bivalent candidate vaccine to control both IB and ND and provides an alternative strategy for the development of cost-effective vaccines for village chickens, especially in the rural areas of developing countries.

## 1. Introduction

Avian Newcastle disease (ND) and infectious bronchitis (IB) caused by the Newcastle disease virus (NDV) and infectious bronchitis virus (IBV) are able to widely spread in chickens and give rise to massive losses in the poultry industry around the world. [1,2,3]. NDV and IBV belong to the family Paramyxoviridae and Coronaviridae, respectively, and the whole genome is approximately 15.2 kb and 27.6 kb in size. NDV and IBV encodes six and four structural proteins, respectively [4,5,6]. Globally, the effective prevention and control of these two important infectious poultry diseases usually depends on a vaccine [7,8]. Both live-attenuated (Gallivac Lasota, Nobilis ND Clone 30) and inactivated vaccines (Gallimune ND, Nobilis Newcavac) are widely administered to poultry, but live vaccines are more commonly used because of their low cost and high effectiveness when applied on a large scale [9]. However, IBV and NDV live-attenuated vaccines likely mutually interfere with concurrent vaccination, which may cause immunity failure [10,11]. Importantly, live vaccines are sensitive to high temperatures, so a cold chain is usually demanded to maintain the quality of the vaccine during transportation and storage [12]. Thus, many commercially available live vaccines are made into lyophilized dosage forms to maintain the biological activity of the virus during long-term storage [13]. Lyophilized vaccines are normally stable prior to reconstitution. Once they have been reconstituted the potency of live-attenuated vaccines can sharply decline [14]. However, the additional refrigeration equipment and cold rooms necessary to maintain the quality of a live vaccine are limited resources for the vaccination of village chickens in rural areas [15]. In general, the costs of distribution and administration are usually greater than the costs of vaccine production, which represents the most prohibitive barrier to extensive immunization implementation, especially in developing countries [16].

Most commercially available NDV vaccines, such as LaSota and the ZJ1 strains, are thermolabile. However, some NDV strains, such as V4, I_2_, and TS09-C, have proven their thermostability [17,18]. Our previous study exchanging the TS09-C strain HN gene into the LaSota strain to obtain a thermostable avirulent recombinant rLS-T-HN strain based on the reverse genetic system (RGS) demonstrated that the HN protein is a crucial determinant of NDV thermostability [12]. During the past two decades, NDV has been proven to be a suitable virus vector for expressing proper exogenous antigens against a variety of pathogens in poultry [19,20,21,22,23]. In a previous study, we developed a recombinant NDV-vector vaccine (rNDV-IBV-T/B) harboring the IBV multiple epitope cassette (S-T/B), which can effectively protect chickens against lethal doses of NDV F48E9 and IBV M41 strain challenges [24]. The S-T/B multiple epitope cassette consists of three neutralizing epitope domains located at the S1 protein 24–61, 132–149, and 291–398 amino acids (AAs) of the IBV Australian T strain (Genbank AAV52771.1) and the four chicken BF2 (major histocompatibility complex class I)-restricted Cytotoxic T lymphocyte (CTL) epitopes located at the S1 protein 413–421 and 517–525 AAs of the QX-like IBV SH1208 strain (Genbank ASA45778.1) and the 45–52 and 413–421 AAs of the Holte strain (Genbank AAA74379.1) [25,26]. Recombinant NDV is an efficient vector for the delivery of IBV-specific immunogens in chickens. Thus, it is practical to develop a recombinant thermostable NDV vector bivalent live vaccine expressing multiple IBV epitopes simultaneously to protect against ND and IB, especially useful developing countries.

In the present study, we designed and rescued a thermostable recombinant NDV bivalent live vaccine harboring the IBV S-T/B cassette and investigated its thermostability, virus titration, and growth kinetics. Furthermore, the protective efficacy of the thermostable recombinant NDV candidate vaccine was evaluated on specific pathogen-free (SPF) chicks subjected to NDV and IBV challenges.

## 2. Materials and Methods

### 2.1. Cells, Viruses, Antibodies, and Ethics Statement

DF-1 and BHK-21 cells were both obtained from the American Type Culture Collection (ATCC, Manassas, VA, USA) and cultured Dulbecco’s modified Eagle’s medium (DMEM, Gibco, Grand Island, NE, USA) supplemented with 10% fetal bovine serum (FBS, Thermo Fisher, Waltham, CA, USA), 100 IU/mL penicillin, and 100 mg/mL streptomycin at 37 °C with 5% CO_2_. Fertile white leghorn SPF embryonated eggs (Beijing Boehringer Ingelheim Vital Biotechnology Co, Ltd. Beijing, China) were used for testing the 50% embryo infectious dose (EID50). The NDV strain F48E9 and IBV M41 strain were both obtained from China Institute of Veterinary Drug Control (Beijing, China). The NDV wildtype isolation strain ZJ1 was the kind gift of Prof. Xiufan Liu (Yangzhou University, China). The recombinant NDV thermostable rLS-T-HN strain and thermolabile LaSota strain were rescued based on the reverse genetic system by Wen et al. and reported previously [12,15]. The rabbit polyclonal antibody against IBV and monoclonal antibody (mAb) against NDV nucleoprotein (NP) were prepared in our laboratory. Horseradish peroxidase (HRP)-conjugated goat anti-rabbit or anti-mouse secondary antibody was purchased from Jackson ImmunoResearch Laboratories (West Grove, PA, USA). The Ethics and Animal Welfare Committee of Shanghai Veterinary Research Institute, China reviewed the all experiments procedures and approval this project (SHVRI-SZ-2020061-02).

### 2.2. Construction of Recombinant NDV Containing a Thermostable HN Gene and IBV S1 Protein Multiple Epitope Cassette

To develop a thermostable NDV bivalent vaccine expressing a multiple IBV epitope cassette, the rLS-T-HN strain was used as a backbone virus vector based on a reverse genetics system according to our previous description [12]. The genomic RNA of previously rescued thermostable recombinant NDV rLS-T-HN strain was used for amplifying the five overlapping cDNA fragments covering the whole genome of NDV through reverse-transcriptase polymerase chain reaction (RT-PCR) and assembled into a pBR322 vector to obtain the full-length cDNA clone named pBR-LS-T-HN. Then, it is linearized at 5′-non-translated region (NTR) of the M gene and ligated with the previously identified IBV S1 protein multiple epitope cassette S-T/B fragment as an additional transcriptional unit using an In-fusion PCR clone kit (Clontech, Mountain View, CA, USA). The constructed plasmid, named pLS-T-HN-T/B, was confirmed by sequencing analysis. Three previous reported helper plasmids pV-NP, pV-P, and pV-L were cotransfected with pLS-T-HN-T/B (at a ratio of 2:1:1:4) into the BHK-21 cells using a Lipofectamine^TM^ 2000 transfection reagent (Thermo Fisher Scientific, Waltham, MA, USA) and incubated at 37 °C for 4 h. Then, the supernatant of the transfected cells was discarded, and the cells were washed twice. The fresh DMEM media culture containing 0.2 µg/mL N-tosyl-L-phenylalanine chloromethyl ketone (TPCK)-trypsin was added and incubated for another 72 h. The recombinant thermostable NDV designated rLS-T-HN-T/B was harvested by freeze-thawing three times and serially passaged for 20 generations (P20) in 10-day-old SPF chicken embryos. The whole genome of the multigenerational rLS-T-HN-T/B strain was analyzed by DNA sequencing (Shanghai Sangon Biotect Co., Ltd., Shanghai, China).

### 2.3. Virus Titration and Growth Kinetics

The virus titration and growth kinetics of the recombinant thermostable rLS-T-HN-T/B strain were carried out according to a previously reported method [27]. The hemagglutination test (HA) assay, EID50 assay, and 50% tissue infectious dose (TCID50) assays were performed in 96-well micro-plates, 10-day-old SPF chicken embryos, and BHK-21 cells respectively to determine the infectivity titration of the recombinant NDV rLS-T-HN-T/B strain, with parental strain LaSota as control. The numeric value of EID_50_ and TCID_50_ were then calculated according to the Reed–Muench reported method [28]. According to the standard protocol (http://www.oie.int/en/standard-setting/terrestrial-manual), the rLS-T-HN-T/B strain was inoculated in one-day-old SPF chickens to investigate the intracerebral pathogenicity index (ICPI) index. Meanwhile, the mean death time (MDT) of rLS-T-HN-T/B strain assay was carried out in 10-day-old SPF chicken embryos.

### 2.4. Antigenicity of the Recombinant rLS-T-HN-T/B Strain

The antigenicity for expressing the multiple epitope cassette S-T/B of the rLS-T-HN-T/B strain was analyzed by Western blotting according to a previously reported method [24].

5 × 10^5^ DF1 cells were seeded in the six-well plate and cultured overnight at 37 °C with 5% CO_2_ condition. A ninety percent confluency of cells was infected at a multiplicity of infection (MOI) of 1 for the first generation (P1), with 20 passages (P20) of rLS-T-HN-T/B and the LaSota virus. Meanwhile, uninfected cells were used as a negative control. Thirty-six hours postinfection, DF1 cells were harvested, lysed with radioimmunoprecipitation assay buffer (RIPA), and centrifuged at 2000 rpm for 10 min (Microfuge 16, Beckman-Coulter, Indianapolis, IN, USA), the supernatant of cytolysis was analyzed by the Western blot. The IBV multiple epitope cassette (S-T/B) was detected by primary antibody rabbit polyclonal sera against IBV, the secondary antibody was an HRP-conjugated goat anti-rabbit antibody. Meanwhile, the NDV NP protein was detected by the primary antibody mouse mAb antibody, and followed by the rabbit antimouse secondary antibody. The bands were visualized using a Pierce ECL Supersignal west pico chemiluminescence kit (Thermo Fisher, Waltham, MA, USA) and imaged using a Tanon automatic image analysis system (Tanon, Shanghai, China). Protein abundance was quantified by densitometric scanning using Image J software (NIH, Bethesda, MD, USA).

### 2.5. Recombinant Virus rLS-T-HN-T/B Detection by Transmission Electron Microscopy (TEM)

To further confirm the morphological characteristics of the thermostable rLS-T-HN-T/B strain, visualization of the thermostable recombinant virus particles and morphological features was performed by TEM detection. The rLS-T-HN-T/B and parental LaSota viruses were purified primarily by sucrose gradient centrifugation according to a previous report [29]. In brief, viral particles collected from the allantoic fluid were purified by ultracentrifugation in a 40–60% (*w*/*w*) sucrose gradient (120,000× *g* for 2 h). The purified viral particles were pelleted by centrifugation at 100,000× *g* under 4 °C for 4 h and resuspended in a phosphate-buffered saline (PBS) solution. Ten microliters of the rLS-T-HN-T/B or LaSota strains were placed onto the shiny side of an electron microscopy grid and then adsorbed for ~5 min. The virus adsorbed three times with 2% uranyl acetate for 45 s and stained the grid promptly. The 98,000× magnification images were visualized by the 80 kV FEI Tecnai Spirit TEM T12 (Thermo Fisher, Waltham, MA, USA).

### 2.6. Assessment of the Thermostability of the rLS-T-HN-T/B Strain

The thermostability of the recombinant NDV rLS-T-HN-T/B strain was investigated according to a previous method [15]. 1.0 mL rLS-T-HN-T/B, LaSota, and ZJ1 strains were added in sealed bottles and then incubated with a 56 °C water bath condition. At the indicated time points, the bottles were chilled in an ice-bath and in order to terminate thermal inactivation and investigate the infectivity titer and HA activity through EID_50_ assay and a standard HA assay, respectively. The decreased infectivity and HA activity of the rLS-T-HN-T/B, ZJ1 and LaSota strains were shown on a logarithmic scale as the heating time increase. Different time points were used for the heat-treatment and HA activity assay of the thermostable and thermolabile viruses. The time points for the thermostable virus were 0, 10, 20, 30, 40, 50, and 60 min, while those for the thermolabile LaSota vaccine and NDV ZJ1 isolation strain were 0, 1, 1.5, 2, and 5 min. In the infectivity titer assay, the heat-treatment time points were all set as 0, 10, 20, 30, 40, 50, and 60 min.

The rLS-T-HN-T/B strain was further preserved (for the long term) at room temperature to evaluate its potential for a thermostable NDV-vector vaccine. Aliquots of undiluted allantoic fluid infected with thermostable rLS-T-HN-T/B or parental LaSota viruses were incubated at 25 °C at 0, 4, 8, 12, and 16 days, respectively, and then the viruses preserved at different time points were titrated in 10-day old embryonated eggs through an EID_50_ assay.

### 2.7. The Protective Efficacy of Thermostable rLS-T-HN-T/B

To estimate the thermostable rLS-T-HN-T/B candidate vaccine’s protective efficacy, 10^6^ EID_50_/_mL_ of the rLS-T-HN-T/B and LaSota vaccines stored at 25 °C for 16 days. Seventy-five one-day-old SPF chicks were randomly divided into six groups (A–F). Among them, groups A, C, and E each included 10 chicks, and groups A and B chicks were vaccinated with rLS-T-HN-T/B via the oculonasal route; meanwhile, groups B, D, and F each included 15 chicks, and group C and D chicks were inoculated with LaSota via the same route.

Groups E and F were treated with 100 µL PBS as the negative control. The chicks were separately housed in negative pressure isolators and feed sterile water and sufficient food. The lethal dose of the NDV F48E9 strain and IBV M41 strain are both the 10^6^ EID_50_ detected by a previous chicken infection study. Three weeks postimmunization, chicks in groups A, C, and E were challenged by NDV and group B, D, and F were inoculated by IBV via the oculonasal route. The clinical symptoms of chicks in all groups were monitored daily for two weeks and the mortality was calculated. Four days postchallenge (DPC), five chicks of groups B, D, and F were euthanized and we carried out tracheal ciliostasis tests.

### 2.8. Serological Assays

To evaluate the antibody responses elicited by the rLS-T-HN-T/B and LaSota vaccines stored at 25 °C for 16 days, ten sera samples were randomly isolated from the wing veins of each vaccinated group 21 days postimmunization. The virus neutralization titer (VN) assays were performed as per our previous description [26]. Zero point one milliliters of each sera two-fold serial dilutions was carried out with PBS and then mixed with the M41 strain at the equal volume of 102 EID50, followed by water bath incubation for 1 h at 37 °C. Zero point two milliliters of sera-virus mixture was inoculated in 10-day-old SPF embryonated eggs via the allantoic cavity route to determine the VN titer. PBS inoculation was the negative control. The mortality and IBV specific lesions of inoculated eggs were observed daily for five days. The VN titers were calculated by the survival embryos in reciprocal of the highest dilution and expressed as the mean ± standard deviation (SD). A hemagglutination inhibition (HI) assay was performed by the previously reported method [30], and the HI titers of the rLS-T-HN-T/B, LaSota vaccine, and PBS groups were expressed as the reciprocal of log2.

### 2.9. Ciliostasis Test

To analyze the protection of the thermostable rLS-T-HN-T/B candidate vaccine against an IBV M41 strain challenge, the ciliary activity of the tracheal explants was examined at 4 DPC. The tracheas of the IBV challenge groups (five chickens in each group) were carefully removed and examined for ciliary activity within 2 h after collection, as described in the literature [31,32]. In brief, ten complete tracheal cross sections about 1–2 mm thick from trachea were prepared and observed with a low-power inverted microscope. The score of ciliary activity was set as four types: all cilia beating represented 0; 75% cilia beating represented 1; 50% cilia beating represented 2; 25% cilia beating represented 3, total ciliostasis represented 4. The ciliostasis score was expressed as the mean ± SD. The higher score means tracheal lack protection and 40 scores indicated totally ciliostasis.

### 2.10. Statistical Analysis

The data were analyzed using one-way ANOVA through GraphPad Prism v8.02 (San Diego, CA, USA) and the results were expressed as the mean ± standard deviation (SD). The significance levels were defined as *p* < 0.05. The survival ratio of the immune protection experiment was determined by log-rank tests.

## 3. Results

### 3.1. The Construction of a Recombinant Thermostable NDV Expressing IBV S1 Protein Multiple Epitope Vaccine (rLS-T-HN-T/B)

**Figure 1:**
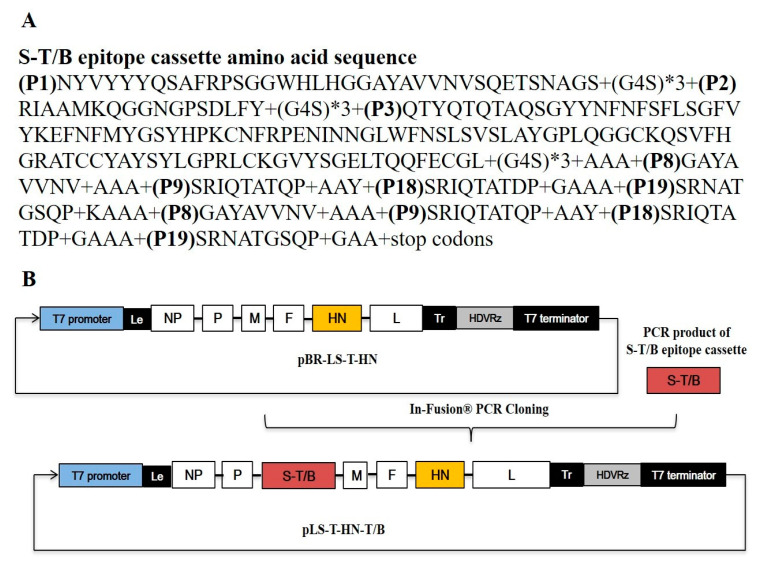
Schematic diagram of the pLS-T-HN-T/B plasmid’s construction. (**A**) The amino acid sequences of the infectious bronchitis virus (IBV) S1 protein T/B multiepitope cassette (S-T/B) contained three neutralizing epitopes (P1, P2, P3) and four BF2-restricted T cell epitopes (P8, P9, P18, P19) linked with flexible amino acids. (**B**) The IBV S-T/B was cloned into the pBR-LS-T-HN vector in the noncoding region downstream from the M gene using the In-Fusion PCR cloning kit, resulting in a pLS-T-HN-T/B clone. Hepatitis delta virus ribozyme (HDVRz) represents the site of the hepatitis delta virus ribozyme sequence. The T7 promoter is indicated by a bold blue box. The orange rectangular box represents the thermostable HN gene; the brick red box represents the IBV S-T/B. Le: leader sequence; Tr: trailer sequence.

To develop the recombinant thermostable NDV bivalent live vaccine expressing IBV-specific immunogens, the previously constructed thermostable NDV rLS-T-HN strain was employed as the vector backbone. Five overlapping cDNA fragments covering the whole genome of the rLS-T-HN strain were amplified by RT-PCR and then assembled into a pBR322 vector to obtain a full-length cDNA clone named pBR-LS-T-HN. The identified IBV multiple epitope cassette S-T/B consisting of three pieces of B-cell-neutralizing epitopes and four pieces of BF2-restricted T cell epitopes was amplified by PCR and ligated with linearized pBR-LS-T-HN using an In-fusion PCR clone kit. A schematic of the recombinant plasmid designated as pLS-T-HN-T/B is shown in Figure 1. Three helper plasmids (pV-NP, pV-P, and pV-L) were cotransfected with pLS-T-HN-T/B into BHK21 cells and then incubated for 72 h at 37 °C with 5% CO_2_. To determine whether the insertion of the IBV multiple epitope cassette affected NDV passage stability, rLS-T-HN-T/B was continuously passaged 20 times through 10-day-old embryonated eggs. The results of the whole genomic sequencing of the rLS-T-HN-T/B stain confirmed that the recombinant NDV was successfully rescued. Furthermore, there were no significant differences in the EID_50_ assay between the first, fifth, tenth, and twentieth passages of the rLS-T-HN-T/B strains, indicating that the infectivity was stable (data not shown).

### 3.2. TEM Detection and Antigenicity Analysis of the Thermostable rLS-T-HN-T/B Strain

**Figure 2:**
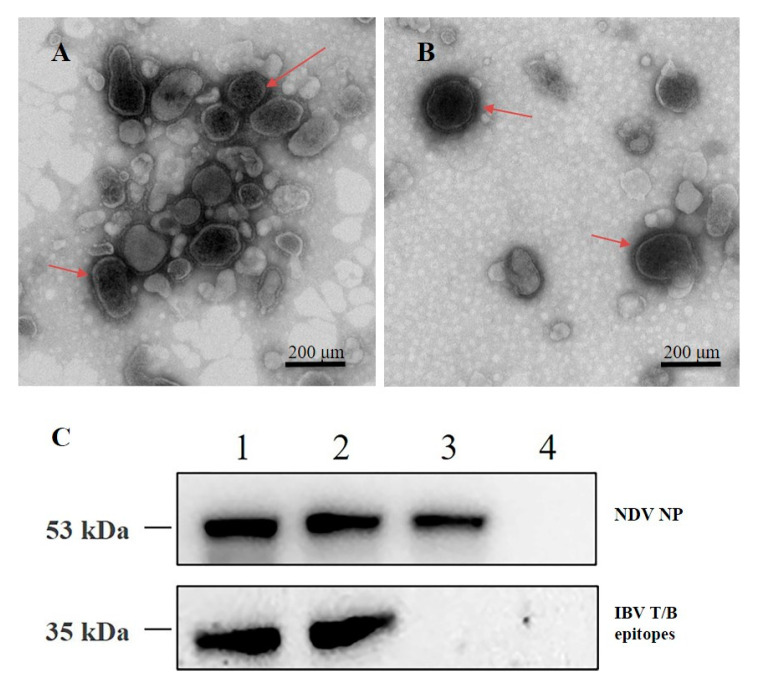
Antigenicity and visualization morphology of the thermostable rLS-T-HN-T/B strain. TEM observation of the thermostable rLS-T-HN-T/B strain (**A**) and thermolabile LaSota strain (**B**), which appear as circles or ovals about 150 nm in diameter. Virions are marked by arrows. Scale bar = 200 nm. (**C**) Western blotting of the rLS-T-HN-T/B and LaSota strains. The IBV-T/B epitope cassette and nucleoprotein (NP) proteins were detected as approximately 35 kDa and 53 kDa bands, respectively. The LaSota strain was only detected as an NP protein band (53 kDa). Lane 1: first generation of the rLS-T-HN-T/B strain; lane 2: 20th generation of the rLS-T-HN-T/B strain; lane 3: LaSota strain; lane 4: uninfected cells control.

To visualize the morphology of the recombinant thermostable rLS-T-HN-T/B virus, the purified virions were negatively stained and analyzed by TEM at 98,000× magnification. The rLS-T-HN-T/B virus appeared as circles or ovals around 150 nm in diameter (Figure 2A). The recombinant NDV envelope structure was located on the outlayer of the viral surface, and this morphology is highly consistent with the parental LaSota strain (Figure 2B). TEM result indicated that rLS-T-HN-T/B strain was successfully rescued.

To analyze the antigenics of the recombinant virus rLS-T-HN-T/B strain, 1 MOI of rLS-T-HN-T/B and the parental strain LaSota were used to infect DF1 cells for 24 h, and the cell lysates were harvested and analyzed by Western blot. The IBV multiple epitope cassette S-T/B protein expressed by the rLS-T-HN-T/B lysates was observed at approximately the 35 kDa band, the image J software was employed to analyze the densitometry readings of the Western blot band and Integrated Density (IntDen) data were 3,998,293 for the first generation and 4,102,153 for the 20th generation rLS-T-HN-T/B strain; meanwhile, the band of the NP protein was located at about 53 kDa, IntDen data were 3,125,881 for the first generation and 3,239,645 for the 20th generation rLS-T-HN-T/B strain,. In contrast, the parental LaSota strain only appeared at the band around the 53 kDa NP protein, and the IntDen was 3,034,471. The uninfected cells control detected no band (IntDen = 0) (Figure 2C). The Western blot results suggested that different passages of recombinant rLS-T-HN-T/B virus could stably express the IBV S/T multiple epitope cassette protein, (original data: Supplementary Materials Figure S1). 

### 3.3. Biological Characterization of the rLS-T-HN-T/B Strain

To determine the biological properties of the recombinant rLS-T-HN-T/B strain, the growth characteristics and pathogenicity were evaluated through virus titration (TCID_50_ or EID_50_), MDT, and ICPI tests. The TCID_50_ titer of rLS-T-HN-T/B (3.5 × 10^7^/mL) was slightly higher than that of LaSota (3.3 × 10^7^/mL) at 48 h postinfection. The EID_50_ titers of the parental LaSota and rLS-T-HN-T/B strains reached 10^9.23^/mL and 10^9.55^/mL, respectively. The HA titers of LaSota and rLS-T-HN-T/B were identical (2^10^). The statistical analysis of the EID_50_, TCID_50_, and HA results indicated that there were no significant differences between rLS-T-HN-T/B and the parental LaSota virus (*p* > 0.05). The MDT of rLS-T-HN-T/B and LaSota were all longer than 120 h, while the ICPI index of rLS-T-HN-T/B was slightly lower (0.03) than that of LaSota (0.05) (Table 1). These results indicated that expression of the IBV multiple epitope cassette did not affect the growth characteristics of the rLS-T-HN-T/B strain. Meanwhile, the MDT and ICPI index suggested that the strain was avirulent.

### 3.4. Determination of the Thermostability of rLS-T-N-T/B

**Figure 3:**
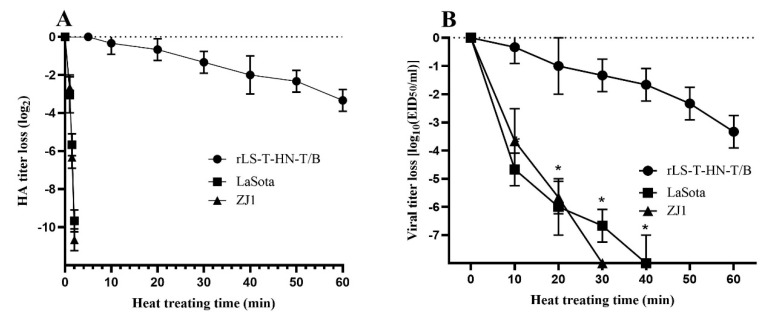
HA activity and infectivity titer thermostability test of the rLS-T-HN-T/B, ZJ1 and LaSota strains. Heat inactivation kinetics were measured by performing an HA assay and EID_50_ assay at the indicated time point at 56 °C. The decreased fractions of HA activity (**A**) and the infectivity titer (**B**) were represented logarithmically as the heating time increased. The mean values and standard deviations are shown for three independent experiments (mean ± SD, *n* = 3). Statistical significance was set at *p* < 0.05, indicated as *.

To investigate thermostability, the rLS-T-HN-T/B and LaSota strains were analyzed in vitro by performing heat treatment at 56 °C and testing the HA activity and infectivity titer. Ultimately, the HA activity of the NDV strains survived longer than 30 min at 56 °C, and these strains required at least 20 min for a 2 log10 decrease in the infectivity titer at 56 °C [33]. The mean time of the HA activity of the LaSota strain and NDV ZJ1 wildtype isolation strain decreased by approximately 4 log2 within 1.5 min, and the HA activity dropped to 0 within 2 min. However, the mean times for the 2 log2 decrease in the HA activity of the rLS-T-HN-T/B strain were around 60 min, which is significantly slower than that of the thermolabile LaSota strain and ZJ1 strain under the 56 °C treatment (*p* < 0.05) (Figure 3A). For the infectivity titer assay, the mean times for the 2 log10 decrease in the EID_50_ of the rLS-T-HN-T/B, ZJ1 and LaSota strains were 40 min and less than 10 min, respectively. The infectivity inactivation rate of the rLS-T-HN-T/B strain was significantly slower than that of the LaSota strain and ZJ1 strain (*p* < 0.05) (Figure 3B). In conclusion, the recombinant rLS-T-HN-T/B strain maintains its thermostability in accordance with the criterion of 56 °C.

To investigate the potency loss of the infectivity titer of the vaccine during storage, the rLS-T-HN-T/B candidate vaccine and LaSota vaccine were stored at 25 °C for 16 days, and the EID_50_ was titrated in 10-day old embryonated eggs at an interval of four days. As shown in Table 2, the rLS-T-HN-T/B strain can be stored at 25 °C for at least 16 days, and the vaccine titer remains stable. However, the infectivity titer of the LaSota strain drops sharply with time under the same preservation conditions. The infectivity titers of the rLS-T-HN-T/B and LaSota vaccines were 6.62 and 1.85 log_10_ EID_50/mL_, respectively, after 16 days of storage. These results demonstrate that the rLS-T-HN-T/B candidate vaccine has highly potent thermostability characteristics.

### 3.5. VN and HI Responses Induced by rLS-T-HN-T/B Candidate Vaccine

To evaluate the efficacy of the thermostable rLS-T-HN-T/B strain stored at 25 °C for 16 days, three weeks postimmunization, sera samples were used to monitor the virus-neutralizing (VN) antibody’s response against IBV and hemagglutination inhibition (HI) against NDV according to the standard protocol of Office International des Epizooties (OIE). As expected, sera from LaSota- and PBS-immunized groups cannot neutralize the IBV stock, the VN titer is 0. Only rLS-T-HN-T/B vaccinated group’s sera elicit a high VN titer (6.78 ± 0.41) against the IBV strain. Considering that the M41 serotype epitope is not included in the recombinant rLS-T-HN-T/B, this result suggested that rLS-T-HN-T/B immunized chicks exhibit the capacity to neutralize heterologous IBV challenge and provide the cross reaction. Sera samples of chickens immunized with rLS-T-HN-T/B, LaSota, and PBS were determined by HI assay, the results were 7.26 ± 0.53, 2.18 ± 0.46, and 0, respectively. Statistical analysis found the rLS-T-HN-T/B group’s HI titer was significantly higher than that of the LaSota and PBS groups (*p* < 0.05) (Table 3). The mean HI titers are expressed as the log2 mean ± SD. This result suggests that vaccines stored at 25 °C for 16 days can significantly affect the HI titer induced by the thermolabile LaSota vaccine. However, the rLS-T-HN-T/B vaccine did not affect the HI titer. Thus, the recombinant rLS-T-HN-T/B strain is thermostable and suitable as a bivalent candidate ND-IB vaccine.

### 3.6. Protective Efficacy of rLS-T-HN-T/B against NDV and IBV Challenges

**Figure 4:**
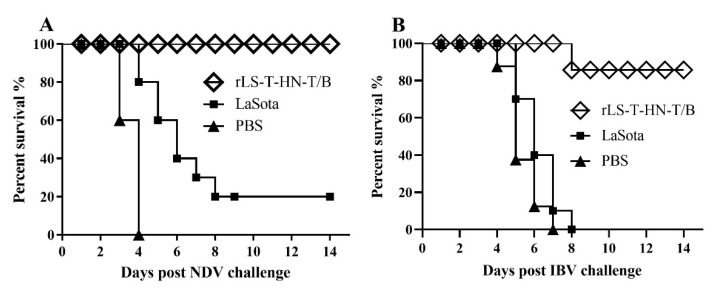
Protective efficacy—16 days at 25 °C preserved thermostable rLS-T-HN-T/B and thermolabile LaSota vaccines. SPF chickens (10 per group) were vaccinated with rLS-T-HN-T/B, LaSota, or PBS. Twenty-one days post-immunization, chickens were challenged with 10^6^ EID_50_ of the NDV F48E9 strain (**A**) and IBV M41 strain (**B**). Clinical symptoms and mortality were monitored daily for 14 days. A comparison of the survival curves based on the log-rank test between the rLS-T-HN-T/B and LaSota strains showed that the two groups have significant differences in their immune protection (*p* < 0.05).

To determine the efficacy of the thermostable rLS-T-HN-T/B vaccine in protecting against IBV and NDV, the rLS-T-HN-T/B and parental LaSota vaccines were stored at 25 °C for 16 days before immunizing the one-day-old SPF chicks via the oculonasal route. Three weeks postimmunization, the chicks of groups A, C, and E were challenged with the NDV F48E9 strain. Group A, immunized with rLS-T-HN-T/B, showed no apparent symptoms post-NDV-challenge. The chicks in PBS control group E showed severe depression, paralysis tremors, and diarrhoea at 2 DPC, and all the chicks died within four days. Group C, inoculated with the long-term room temperature preserved LaSota stain, also manifested listlessness and sneezing, and the chicks appeared to die from four to eight days after the F48E9 virus challenge; the survival rate was 20%. Compared to the chicks immunized with LaSota and PBS, the thermostable rLS-T-HN-T/B immunized group showed a 100% protective ratio, which is significantly higher than that of the LaSota immunization group (20%) (*p* = 0.0035) (Figure 4A).

The chicks in groups B, D, and F were challenged with a heterologous IBV M41 strain 21 days postimmunization, and the clinical symptoms and mortality were recorded for 14 days. Most chickens vaccinated with rLS-T-HN-T/B showed less severe clinical signs post IBV challenge, and only a single chicken died at 9 DPC. In contrast, the chicks in groups D and F, inoculated with PBS and the LaSota vaccine, manifested severe tracheal rales, gasping, and severe respiratory distress; all chickens in these two groups died within eight days post-IBV-challenge (Figure 4B). The survival ratio of the rLS-T-HN-T/B group (90%) was significantly higher than that of the PBS and LaSota vaccinated groups (0%) (*p* = 0.00005).

In total, 16 days of storage at 25 °C significantly weakened the protective efficacy of the LaSota vaccine against NDV challenge. However, the thermostable rLS-T-HN-T/B candidate vaccine was not affected. These results suggest that thermostable NDV helps to store the vaccine at room temperature without affecting the vaccine’s immune effect and can thus help expand the scope of the vaccine’s application, especially for village chickens in rural areas with no cold chain storage environment.

### 3.7. The Tracheal Ciliary Activity of Vaccinated Chickens Post IBV Challenge

The ciliary activity of the tracheal explants of groups B, D, and F was examined at four days post-IBV-challenge. The mean ciliostasis score between the chickens immunized with rLS-T-HN-T/B, LaSota, and PBS control was calculated and expressed as the mean ± SD (Table 3). The mean ciliostasis score of the thermostable rLS-T-HN-T/B vaccine immunized chicks was 4.60 ± 0.81, which is significantly lower than that of the LaSota vaccinated chicks (37.8 ± 1.16) and the PBS control group chicks (39.2 ± 0.74) (*p* < 0.05). The mean ciliostasis score was negatively correlated with vaccine protection efficacy. The lower the score, the stronger the immune protection was against the IBV challenge. The mean ciliostasis scores of the chicks of the LaSota strain and PBS control groups presented no significant differences (*p* > 0.05); all were close to the maximum mean score (40), indicating that these two groups of chicks completely lacked protection against the IBV challenge. Taken together, we conclude that the thermostable rLS-T-HN-T/B vaccine stored at 25 °C for 16 days was still capable of eliciting the necessary immune responses and protecting against IBV invasion.

## 4. Discussion

Avirulent NDV is an ideal vector that has been verified for use as a bivalent live vaccine for the prevention of poultry diseases such as IB, avian influenza, and infectious laryngotracheitis [1,34,35,36]. Due to the high variability of IBV, there are likely multiple serotype specific virulent strains prevalent in the local region, as single-serotype IBV vaccines often lack cross-protective effects [37]. Therefore, a novel vaccine should consider protection against challenges by different serotype IBVs. Our previous studies demonstrated that DNA vaccine or recombinant NDVs expressing the IBV multiple epitope cassette S-T/B, consisting of three pieces of B cell neutralizing epitopes and four pieces of BF2-restricted T cell epitopes, offer potency against homologous and heterologous IBV strain challenges [24,26]. However, the main shortcoming of NDV-vectored bivalent vaccines is that their thermolabile and low temperature characteristics might not be properly maintained during mass vaccination. Thus, vaccine cold-chain equipment (CCE), such as refrigerators and air conditioning systems, are necessities, greatly increasing the cost of vaccination. Indeed, the expenditures for CCE usually exceed the cost of the vaccine itself [38].

As a result, thermolabile live strains are not suitable for vaccinating village chickens in the tropics where temperatures are high and local farmers either lack a sufficient power supply and maintenance capacity or are unable to pay for the cold-chain equipment needed to sustain live thermolabile NDV vaccines [39,40]. Fortunately, some avirulent live NDV strains, such as the I-2 and V4 strains, have been proven to be naturally thermostable and need little to no cold chain equipment, thereby facilitating applications in rural areas where the electricity supply can be extremely deficient [41,42,43,44]. In addition, only a few non-natural thermostable avirulent NDVs were reported in the last decade, such as the TS09-C strain, which was obtained by the serial passage of the V4 strain in BHK-21 cells, and the NDV4-C strain, which was rescued by RGS [45,46].

In our previous study, we developed several chimeric recombinant viruses by exchanging the HN, F, NP, P, and L viral genes between the thermostable TS09-C strain and the thermolabile LaSota strain using RGS. The thermostability tests of these chimeric recombinant NDVs proved that the recombinant chimera NDV (rLS-T-HN) bearing the HN protein derived from the TS09-C strain has a thermostable phenotype, demonstrating that the HN protein of NDV is an important determinant thermostability factor [12]. Furthermore, the developed thermostable recombinant virus rLS-T-HN strain was able to elicit a higher-level HI antibody response than the TS09-C strain and conferred full protection to the chicks against the lethal NDV challenge [12,15].

Based on these results, we developed a recombinant thermostable NDV vector vaccine expressing the IBV multiple epitope cassette S-T/B (rLS-T-HN-T/B). The recombinant rLS-T-HN-T/B strain backbone was derived from the thermostable rLS-T-HN strain. The genetic stability of the P1 and P20 generations of the rLS-T-HN-T/B virus was verified by DNA sequencing. TEM visualization demonstrated that the thermostable rLS-T-HN-T/B strain was successfully rescued and possesses morphology identical to that of the parental LaSota virus. The growth characteristics and pathogenicity tests indicated that rLS-T-HN-T/B was consistent with the LaSota strain. There were no significant differences in the HA, EID_50_, TCID_50_, MDT, and ICPI assays (*p* > 0.05).

The infectivity inactivation rate of the newly developed chimeric recombinant virus rLS-T-HN-T/B with an EID_50_ titer was approximately sevenfold slower than that of LaSota at 56 °C (from 5 to 15 min). Meanwhile, the 2log2 decrease in the HA activity of rLS-T-HN-T/B took more than 15 min, which is 10-fold longer than that of LaSota at 56 °C (from 40 to 60 min). These tests indicate that the rLS-T-HN-T/B strain matches the criteria for the thermostability of NDV [33]. These results are consistent with those of our previous studies [12,15], indicating that the insertion of the IBV multiple epitope cassette in the rLS-T-HN-T/B strain did not affect thermostability. In contrast, Zhao et al. generated chimeric viruses (rNDV4CLaSoP and rLaSoNDV4CP) by exchanging the P protein between the thermostable NDV4-C strain and the thermolabile LaSota strain. The infectivity inactivation rate was evaluated, and the results demonstrated that the NDV4-C strain was thermostable. However, the infectivity of the rLaSoNDV4CP strains dropped sharply, with a loss of the infectivity titer greater than 2 log10 within 20 min, suggesting that the P gene plays an important role in improving thermostability [43]. Furthermore, Liu et al. generated recombinant NDV strains by exchanging the NDV V4 HN gene or by mutating the F gene, revealing that the HN and F proteins could both contribute to the thermostability of NDV [47]. These results will help improve the thermostability of the recombinant NDV, which needs to be explored in further experiments.

The current study showed that 100% and 90% of the bivalent recombinant thermostable rLS-T-HN-T/B candidate vaccine immunized chicks were protected, respectively, against lethal doses of the F48E9 and M41 strains. These protection results are consistent with our previously reported rNDV-IBV-T/B vaccine results [24]. Long-term storage at 25 °C for 16 days at room temperature had no effect on the rLS-T-HN-T/B vaccine. However, the vaccine significantly weakened the protective efficacy of the LaSota vaccine against the NDV challenge, demonstrating that the thermostable rLS-T-HN-T/B vaccine is more reliable for storage and immunization without cold-chain constraints. The literature on this aspect remains scarce for the 16-day thermal exposure under 25 °C of a vaccine followed by a challenge. Omony et al. isolated 168 NDV strains from a waterfowl and live bird market, and 13.7% (23/168) of strains were identified as thermostable [40]. Four thermostable low-virulent isolates were assessed for their potency as NDV vaccine candidates; their 14-day postchallenge protection rates were 60%, 50%, 20%, and 0%, respectively. The mortality protection efficacy of isolates was generally lower than that of the LaSota and I2 vaccines [48]. The results indicate that exchanging thermostable genes between the TS09-C or V4 and LaSota strain using RGS is likely a better strategy for thermostable NDV vaccine development. The ciliary activity test and IBV M41 strain challenge experiment suggested that the potency of the rLS-T-HN-T/B candidate vaccine could cross-protect heterologous IBV. However, we still need to determine whether rLS-T-HN-T/B extensively protects against more heterologous IBV strains in further studies.

## 5. Conclusions

The present study developed a bivalent thermostable NDV expressing multiple IBV epitopes as vaccine candidates to control both IB and ND, which holds promise for improving and extending vaccine storage life with delivery to the chickens through drinking water and a spray without the need for a cold chain. This provides an alternative strategy for the development of a cost-effective vaccine for village chickens, especially in the rural areas of developing countries.

## Figures and Tables

**Table 1:** Biological characterization between rLS-T-HN-T/B and LaSota.

Virus	HA	EID50/mL	TCID50/mL	MDT (h)	ICPI
LaSota	210	9.23	3.2 × 107	>120	0.05
rLS-T-HN-T/B	210	9.55	3.5 × 107	>120	0.03

Abbreviations: HA, hemagglutination titer; EID_50_, 50% egg infectious dose in embryonated eggs; TCID_50_, 50% tissue infectious dose on DF-1 cells; MDT, mean death time in embryonated eggs; ICPI, intracerebral pathogenicity index in one-day-old chickens.

**Table 2:** EID_50_ * titers loss tendency of rLS-T-HN-T/B and LaSota as liquid vaccine storage at 25 °C.

Virus	Titers after Storage for Days				
	0	4	8	12	16
LaSota	9.22 ± 0.42	7.87 ± 0.38	5.82 ± 0.72	2.46 ± 1.22	1.85 ± 0.62
rLS-T-HN-T/B	9.51 ± 0.37	8.82 ± 0.52	8.15 ± 0.46	7.67 ± 0.34	6.62 ± 0.57

* 50% egg infectious dose, log10 per 1 mL. The mean titers were shown from three independent tests (mean ± SD, *n* = 3).

**Table 3:** VN and HI titers of sera sample of immunization chicks.

Immunogen	VN Titer	HI Titer
rLS-T-HN-T/B	6.78 ± 0.41	7.26 ± 0.53
LaSota	0	2.18 ± 0.46
PBS	0	0

The hemagglutination inhibition (HI) titer against NDV and virus neutralization (VN) antibody titer against IBV are expressed as log2 mean ± standard deviation (SD).

## Supplementary Materials

The following are available online at https://www.mdpi.com/2076-393X/8/4/564/s1, Figure S1: Antigenicity of the thermostable rLS-T-HN-T/B strain.
