# Supplementary material for: Development of a Recombinant Thermostable Newcastle Disease Virus (NDV) Vaccine Express Infectious Bronchitis Virus (IBV) Multiple Epitopes for Protecting against IBV and NDV Challenges
Source: Vaccines (Basel). 2020 Oct 1;8(4):564. doi: 10.3390/vaccines8040564 (PMC7712034; doi:10.3390/vaccines8040564)
Supplement: Supplementary file 1 [file vaccines-08-00564-s001.pdf]

Supplemental Materials

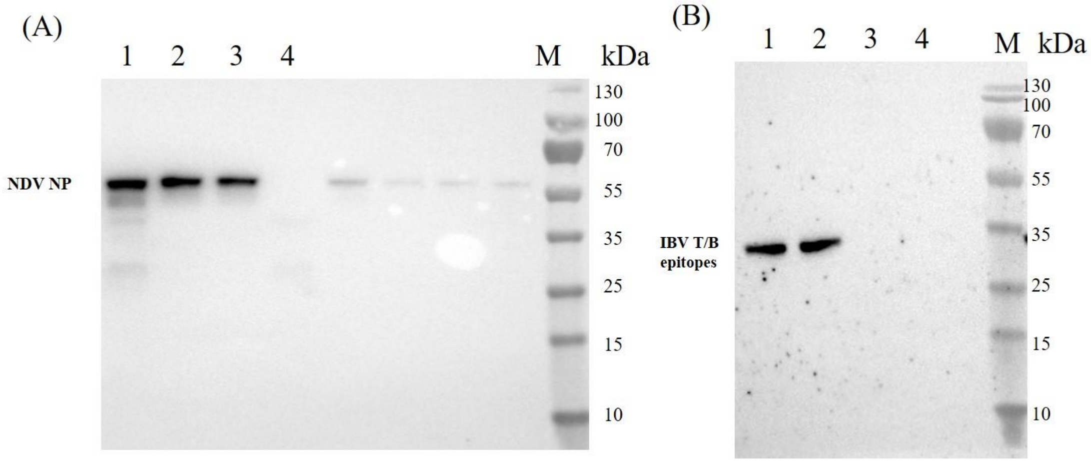

The whole blot (uncropped blots) showing all the bands with all molecular weight markers on the Western in the Supplemental Fig 1: **Antigenicity of the thermostable rLS-T-HN-T/B strain.** Western blot analysis on rLS-T-HN-T/B with NDV NP mAb (A); with IBV polyclonal antibody (B). Lane 1: 1st generation of the rLS-T-HN-T/B strain; Lane 2: 20th generation of the rLS-T-HN-T/B strain; Lane 3: LaSota strain; Lane 4: uninfected cells control.
